# Mental Wellbeing during the Lockdown Period following the COVID-19 Pandemic in Nepal: A Descriptive Cross-sectional Study

**DOI:** 10.31729/jnma.5498

**Published:** 2020-10-31

**Authors:** Carmina Shrestha, Calvin Ghimire, Sajan Acharya, Prabhat KC, Swarndeep Singh, Pawan Sharma

**Affiliations:** 1 Patan Academy of Health Sciences-School of Medicine, Lalitpur, Nepal; 2 New York Medical College/Metropolitan Hospital Center, New York, USA; 3 Department of Psychiatry, All India Institute of Medical Sciences, New Delhi, India; 4 Department of Psychiatry, Patan Academy of Health Sciences-School of Medicine, Lalitpur, Nepal

**Keywords:** *COVID-19*, *lockdown*, *mental wellbeing*, *Nepal*, *pandemic*

## Abstract

**Introduction:**

COVID-19 pandemic has profoundly affected all aspects of society, including mental and physical health. Often missed is the fact that the pandemic is occurring against the backdrop of a very high prevalence of mental health issues. Protecting the mental health of people and healthcare workers is important for long-term positive health outcomes and proper control of the outbreak.

**Methods:**

This is a descriptive cross-sectional, questionnaire-based, online survey by convenience sampling. Ethical approval was obtained from the institutional review committee of Nepal Health Research Council (reference no. 2467). Open access, pre-validated questionnaires were used. Participants with significantly poor Mental wellbeing were identified using the WHO well-being index threshold score. Descriptive statistical analysis was carried out.

**Results:**

Five hundred and fifty-six participants were included in the analysis. Forty percent of the participants reported a WHO well-being index score of below 13, indicative of poor mental wellbeing and a need for further assessment for depression. Poor Mental wellbeing was more prevalent among participants less than 30 years of age, female gender, never married, diagnosed mental disorder, living alone and those using informal sources for COVID-19 related information. More participants with lower sleep quality score and higher perceived stress score reported poor Mental wellbeing.

**Conclusions:**

Combating this challenge requires integration across disciplines. One potential part of the solution is psychological intervention teams. An emerging positive connotation to the pandemic is that it needs to be harnessed as a tool for improving health facilities, community participation, and fighting misinformation.

## INTRODUCTION

The Coronavirus disease 2019 (COVID-19) pandemic has profoundly affected all aspects of society, including mental and physical health. The major psychological stressors during lockdown include duration of isolation, fears of infection, frustration, boredom, inadequate essential supplies, inadequate information, and financial concerns.^1^ The previous SARS epidemic has highlighted the increased incidence of depression, anxiety and various negative psychological effect among survivors.^2^

The inadequacy of preparedness to handle epidemics seems to be a global phenomenon, more pronounced in developing countries. Among the different domains of preparatory measures, ones related to psychological health are the most ignored.^3^ Exploring different factors that correlate to poor mental wellbeing during the lockdown can help guide and advocate for proper and adequate intervention.

The present study was designed to explore mental wellbeing among people in Nepal during the lockdown period following the COVID-19 pandemic.

## METHODS

A descriptive cross-sectional, web-based study, using the CHERRIES checklist^4^ as a guideline was conducted. Convenience sampling was used. The study protocol was approved by the Institutional Review Committee of Nepal Health Research Council (reference no. 2467). The length of the study was 4 months (April 1st to July 30th, 2020). Pre-validated, open-source, questionnaires (World Health Organization Well-Being Index (WHO-5),^5^ Perceived Stress Scale (PSS),^6^ Single item Sleep Quality Scale (SQS))^7^ were used along with additional questions pertaining to demographic parameters. The 5-item World Health Organization Well-Being Index (WHO-5) is a global rating scale measuring subjective well-being. A score of less than or equal to 13 is indicative of poor subjective wellbeing and a need for further evaluation. Perceived Stress Scale categories participants in three groups based on self reported feelings and thoughts during the last month. Score ranging from 0-13 is considered low stress, 14-26 is considered moderate stress, and 27-40 is considered high perceived stress. Single item Sleep Quality Scale directs the respondent to rate the overall quality of sleep (0 = terrible, 1-3 = poor, 4-6 = fair, 7-9 = good, and 10 = excellent) over a 7-day recall period.

The survey was advertised in various social media groups (Facebook, Linkedin, Twitter) using separately created non-identifiable social media accounts at least 3 times during the study period in each. The wording of the announcement was kept neutral with purpose of study, assurance of anonymity and no benefits from participation explicitly stated. Informed consent was obtained from participating volunteers via the same google form used to collect study variables. No identifiable or contact information was obtained from the participants. The data obtained was stored in a password-protected google drive with investigator limited access. Those residing in Nepal were only eligible to participate with a question specifically asking the current city of residence during lockdown. A completeness check during the survey was not performed; however, incomplete questionnaires were omitted in the analysis process. The completion rate was 0.986, 556/564. Participants were allowed to review and change their answers before submission but not after that. Cookies were not used and IP check was not done. A unique site visitor was not ensured but a request to close the questionnaire if the participant thinks s/he has already filled the form was mentioned before informed consent.

The data were analysed using SPSS version 23.0 (Armonk, NY, IBM Corp). Descriptive statistics using mean, standard deviation, frequency and percentage were used to describe the socio-demographic profile, sleep quality, PSS, and WHO well-being index scores of the study participants. The proportion of participants with significantly poor Mental wellbeing were determined using the WHO well-being index threshold score as described in the description of this instrument above.

## RESULTS

Out of 556 participants, there were 283 (50.9%) male and 271 (48.7%) female. The average age of the study sample was 25.93±6.88 years. The majority of the participants were never married 442 (79.5%). The study sample consisted of 254 (45.7%) healthcare workers. All the study participants had received some form of formal education. The detailed socio-demographic profile of the study sample is described (Table 1).

The mean WHO well-being index score representing mental wellbeing was 14.63 ±5.31. A total of 223 (40.1%) participants had a WHO-5 score below 13, indicative of poor mental wellbeing and a need for further assessment for depression. More participants who were less than 30 years 185 (41.6%) reported poor mental well being compared to that of participants more than or equal to 30 years 38 (34.2%). More female participants 127 (46.9%) had poor mental wellbeing compared to that of males 95 (33.6%). The participants who were never married 180 (40.7%) versus the participants who were currently married 43 (39.1%) were comparable in terms of number of participants whose WHO-5 scores indicated poor mental well being. As the education level of participants increased the number of participants reporting poor mental well being decreased (Table 1). Half of the respondents living alone 29 (50.9%) had a WHO 5 wellbeing score indicating poor mental wellbeing versus 173 (39.5%) of people living with their families and 21 (34.4%) people living with at least someone other than immediate family.

**Table 1:** Descriptive profile of study participants (n = 556).

Variables	Total n(%)	WHO-5 ≤13 n(%)	WHO-5 >13 n(%)
**Age (in years)**			
< 30	445 (80.0)	185 (41.6)	260 (58.4)
≥ 30	111 (20.0)	38 (34.2)	73 (65.8)
Gender^*^			
Female	271 (48.7)	127 (46.9)	144 (53.1)
Male	283 (50.9)	95 (33.6)	188 (66.4)
**Marital status**†			
Never Married	442 (79.5)	180 (40.7)	262 (59.3)
Currently Married	110 (19.8)	43 (39.1)	67 (60.9)
**Education**			
SLC, 10 + 2 or equivalent	124 (22.3)	67 (54.0)	57 (46.0)
Bachelor’s degree	304 (54.7)	117 (38.5)	187 (61.5)
Master’s degree or above	128 (23.0)	39 (30.5)	89 (69.5)
**Living Arrangement**			
With family	438 (78.8)	173 (39.5)	265 (60.5)
With other than immediate family	61 (11.0)	21 (34.4)	40 (65.6)
Alone	57 (10.3)	29 (50.9)	28 (49.1)

* Two participants preferred not to report their gender.

† Two participants reported living as legally separated couples, one participant reported to be divorced, and another one to be widowed.

There were an equal number of participants with poor mental wellbeing scores among healthcare workers 99 (39%) and non-healthcare workers 124 (41.1%). Half of the participants with previously diagnosed mental disorder 22 (50.0%) reported poor mental well being versus 201 (39.3%) of participants without any previously diagnosed mental disorder. Information sources like Center for Disease Control, hospital grand rounds, Ministry of Health and Population, Up-to-date, World Health Organization were considered formal sources of information and were used by 99 (34.5%) participants showing poor mental wellbeing. Online news portals, social media and non specific radio or television news were taken as informal sources of information and were used by 124 (46.1%) participants showing poor mental wellbeing (Table 2).

**Table 2:** Mental wellbeing among health workers, participants with mental disorders and users of different sources of information (n = 556).

Variables	Total n(%)	WHO-5 ≤13 n(%)	WHO-5 >13 n(%)
**Health Worker**			
Yes	254 (45.7)	99 (39.0)	155 (61.0)
No	302 (54.3)	124 (41.1)	178 (58.9)
**Diagnosed Mental Disorder**			
Yes	44 (7.9)	22 (50.0)	22 (50.0)
No	512 (92.1)	201 (39.3)	311 (60.7)
**Main source of COVID-19 related information**			
Formal Source	287 (51.6)	99 (34.5)	188 (65.5)
Informal Source	269 (48.4)	124 (46.1)	145 (53.9)

The mean Sleep Quality Scale score of the total participants was 7.35±2.10. Better the sleep score, less was the number of participants reporting poor mental wellbeing (Table 3). The mean Perceived Stress Scale score of the total participants was 17.54±6.29. Thirty-one (83.8%) participants with high perceived stress score also reported poor mental well being versus 179 (46.1%) participants with moderate stress score and 13 (9.9%) participants with low stress score.

**Table 3:** Mental wellbeing and SQS and PSS score of study participants (n = 556).

Variables	Total n(%)	WHO-5 ≤13 n(%)	WHO-5 >13 n(%)
Sleep quality score			
Terrible (0)	3 (0.5)	3 (100.0)	-
Poor (1-3)	29 (5.2)	25 (86.2)	4 (13.8)
Fair (4-6)	127 (22.8)	78 (61.4)	49 (38.6)
Good (7-9)	304 (54.7)	94 (30.9)	210 (69.1)
Excellent (10)	93 (16.7)	23 (24.7)	70 (75.3)
PSS Score			
Low Stress (0-13)	131 (23.6)	13 (9.9)	118 (90.1)
Moderate Stress (14-26)	388 (69.8)	179 (46.1)	209 (53.9)
High Stress (27-40)	37 (6.7)	31 (83.8)	6 (16.2)
PSS: Perceived stress scale.			

## DISCUSSION

The ongoing novel coronavirus outbreak, COVID-19 which emerged in Wuhan, Hubei province, China, has now spread worldwide. As of September 4, 2020, the world has witnessed 865,154 deaths due to COVID-19.^8^ In Nepal, there are 44,236 diagnosed cases of COVID-19 of which 25,561 have recovered and 271 have died.^9^ Beginning in August, Centre of Disease Control issued a level 3 warning for Nepal — avoid all nonessential travel.^10^ COVID-19 being a fairly novel virus, the data is currently scarce. Yet, studies conducted in various affected countries suggest significant mental distress secondary to it.

Mental wellbeing includes psychological functioning, as well as cognitive and emotional dimensions of wellbeing.^11^ The World Health Organization has declared positive mental health to be the ‘foundation for well-being and effective functioning for both the individual and the community’ and defined it as a state ‘which allows individuals to realize their abilities, cope with the normal stresses of life, work productively and fruitfully, and make a contribution to their community.’ The capacity for mutually satisfying and enduring relationships is another important aspect of positive mental health.^3^ Stress classically refers to “the bodily processes that result from circumstances that place physical or psychological demands on an individual".^12^ An increase in perceived stress during a pandemic is a well-known phenomenon.^13^

There is ample evidence that the direct and indirect psychological and social effects following epidemics are pervasive and could affect current and future mental health. Deaths by suicide increased in the USA during the 1918-19 influenza pandemic and among older people in Hong Kong during the 2003 severe acute respiratory syndrome (SARS) epidemic.^14,15^ Often missed is the fact that the pandemic is occurring against the backdrop of a very high prevalence of mental health issues.^16^ The potential fallout of the economy, separation from friends and family, boredom, loss of freedom, and uncertainty of situation are likely to have profound effect on mental health.^1^ The consequences could include suicide, self-harm, alcohol and substance misuse and domestic violence.^1^

Although widely accepted by various experts,^2,6^ there isn't a globally accepted consensus regarding the short and long term effects of pandemics on mental health. During the SARS-CoV-1 epidemic, people had psychiatric symptoms upto months after the control of the epidemic^17^ which persisted even a year after the outbreak.^16^ Furthermore, perceived stress among healthcare workers during the SARS^16^ and COVID-19^18^ outbreaks were higher. However, another longitudinal study by Wang et al during COVID-19 pandemic showed no difference in depression, anxiety and stress during the initial phase of COVID-19 and a month later.^19^ This shows that our current understanding of the psychological impact of epidemic is inadequate and indicates the need for further studies.

A study conducted by Sonderskov et al., 2020 in Denmark suggested worsening of mental health as compared to before the pandemic.^20^ Although comparison of this study with that of Nepal is limited by differences in baseline mental health issues, cultural and socioeconomic background, the conclusion of the study resonates with that indicated by ours. Studies with proportionate distribution of demographic characters have pointed out various risk factors for poor mental health, especially depressive and anxiety disorders. The major risk factors identified were female gender,^21^ current or past medical history,^22^ and poor-self-related health.^23^ These findings remain consistent with our study.

Our study demonstrated that those with previously diagnosed mental illness were more likely to report poor mental wellbeing. This finding is supported by studies conducted by Fernandez-Aranda et al^24^ and Zhou et al^25^ who report exacerbation of psychiatric symptoms in patients with pre-existing psychiatric illness.

Frontline healthcare workers in Wuhan reported more severe anxiety, depressive symptoms and insomnia.^26^ Our study; however, showed no difference in prevalence of poor mental wellbeing among healthcare workers and the general population. Healthcare workers form a vulnerable population during outbreaks with increased risk of exposure to infection and job burnout. The inconsistency in our study could be explained by the limited study sample and initial phase of COVID-19 in Nepal with limited case burden.

Our study, with a mean age of participation of 25 years, demonstrated that poor mental wellbeing was more prevalent in the younger age group. Similar finding was reported in a study conducted in China, where adolescents had a higher incidence of depressive symptoms during COVID-19 than adults.^27^

In our study, participants primarily using informal sources (Facebook, other social media) to obtain information on COVID-19 were more likely to report poor mental wellbeing. Information on COVID-19 is ever evolving. Additionally, myths and misinformation regarding the epidemic is likely to aggravate anxiety about becoming infected and uncertainty about the future.^28^ In a study conducted in Italy to assess sleep quality following COVID-19, 17.4% participants reported moderate/severe insomnia. Participants with chronic conditions and females were more vulnerable to sleep disturbances.^29^ Sleep quality score was found to be an important predictor of mental wellbeing in our study.

Our study showed that participants living alone were more prevalent to have poor Mental wellbeing than those living with family or friends. Cao W, similarly, reported increased anxiety among college students who were living alone during this pandemic.^30^ Gao et al.^31^ and Mazza et al.^32^ reported lower educational status being a significant factor for development of depression and anxiety during this pandemic. These findings remain consistent with our study where more participants with higher education levels reported good mental wellbeing.

In our study, 40.1% of the participants had a WHO well-being index indicating poor Mental wellbeing. This translates to increased need for psychiatric consultation and treatment during and after the pandemic. One potential part of the solution is psychological intervention teams. It is a tried and tested method of providing psychological help during pandemics; the latest example of which was seen in the RenMin Hospital of Wuhan University and Mental Health Center of Wuhan.^33^ The team is composed of hospital managers and psychiatrists and are responsible for (a) formulating psychological intervention materials and rules, (b) providing technical guidance and supervision (c) participating in clinical psychological intervention for healthcare workers and patients, and (d) providing telephone guidance to help deal with mental health problems.^33^

## CONCLUSIONS

Our study demonstrated that poor mental wellbeing was more prevalent among participants of younger age, female gender, never married, living alone, diagnosed mental disorders and those using informal sources for COVD-19 related information. Participants with poor sleep scores, higher perceived stress scores were also more likely to report poor mental wellbeing. More studies done on a larger scale are needed to determine accurate correlates to mental wellbeing in order to formulate effective interventions and target vulnerable populations.

Combating this challenge requires integration across disciplines and incorporation of lessons learnt from past epidemics. In situations where lockdown is the most effective and necessary means to control a pandemic, officials should enforce lockdown for no longer than required, provide a clear rationale for lockdown, and ensure the reasonable needs of people in lockdown are met. In order to deal with uncertainty caused by misinformation, community participation can be mobilized to share accurate information about the COVID-19 pandemic through authorized media channels.

## References

[1] Holmes EA, O'Connor RC, Perry VH, Tracey I, Wessely S, Arseneault L (2020). Multidisciplinary research priorities for the COVID-19 pandemic: a call for action for mental health science. Lancet Psychiatry.

[2] Lee SA (2020). Coronavirus Anxiety Scale: A brief mental health screener for COVID-19 related anxiety. Death Stud.

[3] Satici B, Saricali M, Satici SA, Griffiths MD (2020). Intolerance of Uncertainty and Mental wellbeing: Serial Mediation by Rumination and Fear of COVID-19. Int J Ment Health Addict.

[4] Eysenbach G (2004). Improving the Quality of Web Surveys: The Checklist for Reporting Results of Internet E-Surveys (CHERRIES). J Med Internet Res.

[5] WHO (1998). Wellbeing Measures in Primary Health Care/The Depcare Project.

[6] Cohen S, Kessler RC, Underwood Gordon L (1995). Perceived stress scale. Measuring Stress: A Guide for Health and Social Scientists.

[7] Snyder E, Cai B, DeMuro C, Morrison MF, Ball W (2018). A New Single-Item Sleep Quality Scale: Results of Psychometric Evaluation in Patients With Chronic Primary Insomnia and Depression. J Clin Sleep Med.

[8] World Health Organization (2020). WHO Coronavirus Disease (COVID-19) Dashboard [Internet]. Global Situation.

[9] Ministry of Health and Population Nepal (2020). COVID-19 Dashboard [Internet]. Nepal COVID Update.

[10] Centers for Disease Control and Prevention (2020). COVID-19 in Nepal [Internet]. Travelers' Health.

[11] Brooks SK, Webster RK, Smith LE, Woodland L, Wessely S, Greenberg N (2020). The psychological impact of quarantine and how to reduce it: rapid review of the evidence. Lancet.

[12] Tennant R, Hiller L, Fishwick R, Platt S, Joseph S, Weich S (2007). The Warwick-Edinburgh Mental wellbeing Scale (WEMWBS): development and UK validation. Health Qual Life Outcomes.

[13] Georgiou N, Delfabbro P, Balzan R (2020). COVID-19-related conspiracy beliefs and their relationship with perceived stress and pre-existing conspiracy beliefs. Personal Individ Differ [Internet].

[14] Sowden GL, Huffman JC (2009). The impact of mental illness on cardiac outcomes: a review for the cardiologist. Int J Cardiol.

[15] Cheung YT, Chau PH, Yip PS (2008). A revisit on older adults suicides and Severe Acute Respiratory Syndrome (SARS) epidemic in Hong Kong. Int J Geriatr Psychiatry.

[16] Lee AM, Wong JG, McAlonan GM, Cheung V, Cheung C, Sham PC (2007). Stress and psychological distress among SARS survivors 1 year after the outbreak. Can J Psychiatry.

[17] Peng EY, Lee MB, Tsai ST, Yang CC, Morisky DE, Tsai LT (2010). Population-based post-crisis psychological distress: an example from the SARS outbreak in Taiwan. J Formos Med Assoc.

[18] Babore A, Lombardi L, Viceconti ML, Pignataro S, Marino V, Crudele M (2020). Psychological effects of the C0VID-2019 pandemic: Perceived stress and coping strategies among healthcare professionals. Psychiatry Res.

[19] Wang C, Pan R, Wan X, Tan Y, Xu L, McIntyre RS (2020). A longitudinal study on the mental health of general population during the COVID-19 epidemic in China. Brain Behav Immun.

[20] Sonderskov KM, Dinesen PT, Santini ZI, Ostergaard SD (2020). The depressive state of Denmark during the COVID-19 pandemic. Acta Neuropsychiatr.

[21] Malhi GS, Mann JJ (2018). Depression. Lancet.

[22] Kohler CA, Evangelou E, Stubbs B, Solmi M, Veronese N, Belbasis L (2018). Mapping risk factors for depression across the lifespan: An umbrella review of evidence from meta-analyses and Mendelian randomization studies. J Psychiatr Res.

[23] Molarius A, Janson S (2002). Self-rated health, chronic diseases, and symptoms among middle-aged and elderly men and women. J Clin Epidemiol.

[24] Fernandez-Aranda F, Casas M, Claes L, Bryan DC, Favaro A, Granero R (2020). COVID-19 and implications for eating disorders. Eur Eat Disord Rev.

[25] Zhou J, Liu L, Xue P, Yang X, Tang X (2020). Mental Health Response to the COVID-19 Outbreak in China. Am J Psychiatry.

[26] Lai J, Ma S, Wang Y, Cai Z, Hu J, Wei N (2020). Factors Associated With Mental Health Outcomes Among Health Care Workers Exposed to Coronavirus Disease 2019. JAMA Netw Open.

[27] Wang C, Pan R, Wan X, Tan Y, Xu L, Ho CS (2020). Immediate Psychological Responses and Associated Factors during the Initial Stage of the 2019 Coronavirus Disease (COVID-19) Epidemic among the General Population in China. Int J Env Res Public Health.

[28] Zandifar A, Badrfam R (2020). Iranian mental health during the COVID-19 epidemic. Asian J Psychiatr.

[29] Gualano MR, Lo Moro G, Voglino G, Bert F, Siliquini R (2020). Effects of Covid-19 Lockdown on Mental Health and Sleep Disturbances in Italy. Int J Env Res Public Health.

[30] Cao W, Fang Z, Hou G, Han M, Xu X, Dong J (2020). The psychological impact of the COVID-19 epidemic on college students in China. Psychiatry Res.

[31] Gao J, Zheng P, Jia Y, Chen H, Mao Y, Chen S (2020). Mental health problems and social media exposure during COVID-19 outbreak. PLoS One.

[32] Mazza C, Ricci E, Biondi S, Colasanti M, Ferracuti S, Napoli C (2020). A Nationwide Survey of Psychological Distress among Italian People during the COVID-19 Pandemic: Immediate Psychological Responses and Associated Factors. Int J Env Res Public Health.

[33] Kang L, Li Y, Hu S, Chen M, Yang C, Yang BX (2020). The mental health of medical workers in Wuhan, China dealing with the 2019 novel coronavirus. Lancet Psychiatry.

